# Precision Dermatology: A Review of Molecular Biomarkers and Personalized Therapies

**DOI:** 10.3390/cimb46040186

**Published:** 2024-03-30

**Authors:** Isabella J. Tan, Alicia Podwojniak, Aarushi Parikh, Bernard A. Cohen

**Affiliations:** 1 Rutgers Robert Wood Johnson Medical School, 125 Paterson Steet, New Brunswick, NJ 08901, USA; ijt11@rwjms.rutgers.edu (I.J.T.); akp146@rwjms.rutgers.edu (A.P.); 2 Rowan-Virtua School of Osteopathic Medicine, 113 E Laurel Road, Stratford, NJ 08084, USA; podwoj79@rowan.edu; 3 Department of Dermatology, The Johns Hopkins Hospital, Baltimore, MD 21287, USA

**Keywords:** personalized dermatology, biomarker-driven therapies, molecular insights

## Abstract

The evolution of personalized medicine in dermatology signifies a transformative shift towards individualized treatments, driven by the integration of biomarkers. These molecular indicators serve beyond diagnostics, offering insights into disease staging, prognosis, and therapeutic monitoring. Specific criteria guide biomarker selection, ensuring attributes like specificity, sensitivity, cost feasibility, stability, rapid detection, and reproducibility. This literature review, based on data from PubMed, SCOPUS, and Web of Science, explores biomarkers in Hidradenitis Suppurativa (HS), Psoriasis, Atopic Dermatitis (AD), Alopecia Areata (AA), Vitiligo, and Chronic Spontaneous Urticaria (CSU). In HS, TNF-α, IL-1β, and MMPs serve as biomarkers, influencing targeted therapies like adalimumab and anakinra. Psoriasis involves biomarkers such as TNF-α, IL-23, and HLA genes, shaping treatments like IL23 and IL17 inhibitors. AD biomarkers include ECP, IL-4, IL-13, guiding therapies like dupilumab and tralokinumab. For AA, lipocalin-2, cytokines, and genetic polymorphisms inform JAK inhibitors’ use. Vitiligo biomarkers range from cytokines to genetic markers like TYR, TYRP1, guiding treatments like JAK inhibitors. CSU biomarkers encompass IgE, cytokines, and autologous serum tests, influencing therapies like omalizumab and cyclosporine. Comparing conditions, common proinflammatory markers reveal limited specificity. While some biomarkers aid diagnosis and standard treatments, others hold more scientific than clinical value. Precision medicine, driven by biomarkers, has shown success in skin malignancies. Future directions involve AI-powered algorithms, nanotechnology, and multi-omics integration for personalized dermatological care.

## 1. Introduction

The evolution of personalized medicine within dermatology marks a pivotal shift toward tailored treatments, catalyzed by the integration of biomarkers. These molecular signposts serve as indicators, delineating normal biological processes, aberrant pathways, and therapeutic responses. Their role extends far beyond diagnostic tools, giving insights into disease staging, prognostication, and monitoring therapeutic efficacy.

Central to their utility is the criterion for biomarker selection, ensuring specificity, sensitivity, cost feasibility, stability, rapid detection, and reproducibility. Ensuring biomarker selection encompasses specificity, sensitivity, cost feasibility, environmental stability, swift detectability, and reproducibility stands as paramount criteria within this landscape. Within dermatology, these markers illuminate complex conditions, allowing for nuanced understanding and targeted interventions. Common biomarkers conventionally include interleukins (IL), chemokine (C-C motif) ligands, human leukocyte antigen (HLA) markers, and proteins such as matrix metalloproteinases (MMPs), eosinophil cationic protein (ECP), thymic stromal lymphopoietin (TSLP), and C-reactive protein (CRP) [1,2,3].

In addition, chronic dermatoses represent a diverse group of persistent skin conditions that often require long-term management strategies. These conditions, such as psoriasis, eczema, acne vulgaris, and rosacea, can significantly impact patients’ quality of life due to their recurrent nature and associated symptoms. Monitoring and treating chronic dermatoses necessitate a comprehensive approach that considers the underlying pathophysiology, disease severity, patient preferences, and response to therapy.

Monitoring chronic dermatoses typically involves regular assessments of disease activity, symptom severity, and treatment response. Clinicians employ a variety of tools and scoring systems to objectively measure disease severity and track changes over time. For instance, in psoriasis, the Psoriasis Area and Severity Index (PASI) is commonly used to quantify the extent and severity of skin involvement, while the Eczema Area and Severity Index (EASI) is utilized in eczema to assess disease severity based on erythema, induration, excoriation, and lichenification.

In addition to clinical assessments, monitoring chronic dermatoses often involves evaluating biomarkers and surrogate endpoints to provide insights into disease activity and treatment efficacy. Biomarkers, such as cytokines, chemokines, and inflammatory mediators, can serve as indicators of underlying inflammatory processes and may help guide treatment decisions. Many skin diseases involve elevated levels of interleukin-17 (IL-17) and tumor necrosis factor-alpha (TNF-alpha), which induce pathways like AKT/beta-catenin implicated in disease pathogenesis [4]. This prompts the development of targeted biologic therapies that specifically inhibit these cytokines. In recent years, there has been growing interest in personalized medicine approaches for the management of chronic dermatoses, driven by advancements in biomarker research and therapeutic targeting. Tailoring treatment strategies based on individual patient characteristics, such as disease phenotype, genetic predisposition, and biomarker profiles, holds promise for optimizing therapeutic outcomes and minimizing adverse effects. However, challenges remain in the effective management of chronic dermatoses, including the need for improved biomarkers to accurately predict treatment response and disease progression. Additionally, access to specialized therapies, cost considerations, and adherence to long-term treatment regimens pose barriers to optimal care for some patients.

The applicability of biomarkers not only involves its use in chronic dermatoses, but has crucial utility in a broader sense, acting as biological measurements aiding in early efficacy and safety evaluations, also spanning various applications, from disease detection to assessing health status. Their roles encompass diagnostic precision, staging diseases, prognosticating outcomes, and even predicting and monitoring responses to interventions. Navigating the landscape of biomarkers involves comprehending clinical endpoints, which serve as reflections of patient experiences, functionalities, and survival. Further complicating this terrain are surrogate endpoints, biomarkers intended to substitute for clinical endpoints, anticipated to predict clinical benefits based on scientific evidence.

Within the discussion of personalized medicine within dermatology, the comprehensive understanding and utilization of biomarkers, clinical endpoints, and surrogate endpoints emerge as fundamental pillars shaping the future of tailored therapeutic interventions [5].

## 2. Methods

The literature review was conducted using PubMed, SCOPUS, and Web of Science databases, employing the NLM Medical Subject Heading (MeSH) to optimize search term selection. The search strategy involved the formulation of strings such as (“biomarkers” OR “serum marker”) AND the relevant disease, tailored to target specific conditions of interest. Inclusion criteria encompassed articles presenting primary data from randomized controlled trials, cohort studies, retrospective studies, case studies, or case series, published within the preceding five years, and focused on biomarkers associated with various dermatological conditions including hidradenitis suppurativa (HS), psoriasis, atopic dermatitis (AD), alopecia areata (AA), vitiligo, or chronic spontaneous urticaria (CSU). Priority was given to primary sources, while secondary sources such as reviews were consulted to complement potential gaps in information. Exclusion criteria comprised abstracts, articles lacking full-text availability, ongoing studies, and publications not in English. Two reviewers conducted full-text appraisal, evaluating each article for relevance and adherence to proper data reporting standards. Additionally, any pertinent articles identified beyond the initial search terms were integrated as necessary to ensure comprehensive coverage of the subject matter.

## 3. Biomarkers in Dermatological Conditions

### 3.1. Hidradenitis Suppurativa

Hidradenitis Suppurativa (HS), a chronic inflammatory skin disorder, has increasingly drawn attention due to its debilitating impact on patients’ quality of life. Characterized by recurrent, painful nodules and abscesses in intertriginous areas such as the axillae, groin, and buttocks, HS often leads to severe pain, scarring, and significant psychosocial distress for affected individuals.

Recent studies have shown the role of specific biomarkers in uncovering the pathophysiology of HS and paving the way for targeted therapeutic interventions. These biomarkers include a variety of proinflammatory cytokines, chemokines, and inflammatory mediators implicated in the dysregulated immune response and follicular occlusion characteristic of HS lesions.

Tumor necrosis factor-alpha (TNF-α), interleukin-1 beta (IL-1β), and matrix metalloproteinases (MMPs) have shown potential significance in HS pathogenesis, highlighting the immune response dysregulation and tissue remodeling characteristic of HS [6]. These biomarkers may indicate disease severity and progression, which can guide therapeutic strategies.

The introduction of targeted therapies in HS has significantly developed, largely influenced by biomarker insights. Clinical trials involving biologics targeting TNF-α, such as adalimumab, have demonstrated promising results in reducing HS lesion count [7]. Similarly, a recent study examined the efficacy of adalimumab in HS patients, revealing a substantial reduction in inflammatory lesion count and improved quality of life scores over a 12-week period [8]. Anti-IL-1-directed therapies have also shown promise. A randomized clinical trial explored the efficacy of anakinra, an IL-1 antagonist, demonstrating a significant decrease in inflammatory lesions and pain scores in HS patients compared to the control [9].

In a systematic review evaluating 48 discrete HS biomarkers, only 1 diagnostic (serum IL-2R), 1 monitoring (dermal Doppler vascularity), and 2 predictive biomarkers (epithelialized tunnels and positive family history of HS) achieved significantly scaled ratings, which may inform future research into HS biomarker validation, potentially steering targeted therapies and allowing for more effective and personalized interventions [10].

### 3.2. Psoriasis

Psoriasis (PsO) is a chronic autoimmune skin condition characterized by the rapid buildup of skin cells, leading to the formation of thick, silvery scales and itchy, dry patches. While its exact cause remains unknown, the immune system’s abnormal response is believed to play a crucial role. Researchers have been diligently working to identify biomarkers associated with PsO, aiming to enhance diagnostic accuracy and refine treatment strategies.

The pathogenic Tumor Necrosis α (TNF-α)/Interleukin(IL)-23/IL-17 has been shown to have a central role in the PsO inflammatory cascade [11]. Additional key biomarkers that have been studied include HLA-C06:02, HLA-B27, HLA-B38, HLA-B*08, CXCL10, Mac-2 binding protein, integrin b5, matrix metalloproteinase-3, macrophage-colony stimulating factor, tyramine, and mucic acid [12]. The subsequent biomarkers have also been identified across various domains to predict disease severity: LCE3D, IL23R and IL23A, the NFKBIL1 loci, HLA-C06:02, IL-17A, IgG aHDL, GlycA, I-FABP, kallikrein 8, and tyramine [11]. In essence, most of these biomarkers are associated with the inflammatory response, and many are associated with inflammatory-related comorbidities, such as cardiovascular disease [13].

As a result, drugs that inhibit the IL-23/IL-17 pathway have shown great promise in treating PsO. Clinical trials with IL23 inhibitors (ustekinumab, guselkumab, tildrakizumab, risankizumab) and IL17 inhibitors (secukinumab, ixekizumab, brodalumab) demonstrate remarkable and varying degrees of improvement [14]. Inhibitors of other upstream molecules involved in IL-17 and/or IL-23 signaling are also being investigated, such as those from the Janus kinase/signal transducers and activators of transcription (JAK/STAT) pathways. One clinical study found that TYK2/Janus kinase 1 inhibitor PF-06700841 reduced IL-17A and IL-17F mRNA and improved PsO clinical symptoms [15]. Understanding the genetic basis of psoriasis allows clinicians to consider individualized treatment plans based on a patient’s unique genetic profile.

### 3.3. Atopic Dermatitis

Atopic dermatitis (AD) is a chronic and inflammatory skin condition characterized by red, itchy rashes. Common symptoms also include dry and scaly skin, and individuals with AD may experience flare-ups triggered by factors such as stress or exposure to certain allergens. The exact cause of AD is believed to involve a combination of genetic, immune system, and environmental factors.

A number of relevant biomarkers have been linked specifically with AD. Eosinophil cationic protein (ECP) [16], released by eosinophils, exhibits antimicrobial properties and has shown to serve as a biomarker in AD, indicating eosinophil activation and contribution to the local inflammatory response in the skin, leading to the characteristic redness, swelling, and itching seen in AD. Cytokines interleukin (IL)-4 and IL-13 [17] are also linked with AD, as they mediate Type 2 inflammation, the distinctive immune response whereby T-helper 2 (Th2) cells orchestrate an allergic cascade, prompting the recruitment of eosinophils, mast cells, and other immune effectors; the heightened Type 2 immune response contributes to the characteristic features of skin inflammation, pruritus, and compromised barrier function of AD. Thymic stromal lymphopoietin (TSLP) [18] which is produced by epithelial cells, has also shown to be crucial for initiating allergic responses and in the pathogenesis of AD. The filaggrin gene (FLG) as well predisposition to AD [19]. FLG is crucial for the skin’s barrier function, encoding filaggrin, which plays an essential role in maintaining the integrity of the stratum corneum; this layer acts as a protective barrier, preventing the loss of moisture and shielding the body from environmental factors, irritants, and allergens. Its role in AD may include the development of dry and scaly skin. Periostin, which induces TSLP production from keratinocytes [20] is implicated in AD pathogenesis too. IL-31 [21], another cytokine, also possibly contributes to the persistent itching experienced by individuals with AD, further exacerbating skin inflammation and discomfort. Most of these markers are related to the epithelial barrier, TH2 cytokines, and immune cells such as mast cells, eosinophils and dendritic cells.

Biomarkers associated with clinical severity of AD encompass indicators linked to general inflammation, such as serum lactate dehydrogenase and C-reactive protein, which indicate a systemic inflammatory response in individuals with the condition. The presence of CCL17/thymus and activation-regulated chemokine (TARC) provide further insights into the role of allergic inflammation in AD. CCL26/eosinophil-attracting chemokine (eotaxin-3), CCL27/CTACK, CCL18/pulmonary and activation-regulated chemokine, and CCL22/macrophage-derived chemokine (MDC) [22] signify increased migration of T-cells, eosinophils, and macrophages to the skin, insinuating how the immune dysregulation is a major driver of the AD.

AD presents therapeutic challenges due to its multifactorial origins. However, significant progress has been achieved in therapeutic approaches, particularly with the development of biologic cytokine and receptor antagonists, as well as JAK inhibitors. For example, dupilumab, a fully human monoclonal antibody that inhibits the activity of interleukin-4 and interleukin-13, has demonstrated effectiveness in individuals with asthma and heightened eosinophil levels [23] and has continued to prove its efficacy in patients [24]. Anti-IL-13 tralokinumab has also demonstrated both early and enduring enhancements in the signs and symptoms of AD [25]. In an extension study of two clinical trials, baricitinib, an oral JAK inhibitor, demonstrated long-term efficacy in patients with moderate to severe AD [26]; the decision to use it may often be based on a patient’s previous response to systemic therapies.

The IL-31 pathway has also become a focal point for novel treatments aiming to address immunologic dysfunction and restore the compromised skin barrier in AD patients. Nemolizumab, a monoclonal antibody targeting IL-31RA, shows promise in reducing pruritus, inflammation, and aiding skin barrier recovery in many clinical trials [27]. Individuals with elevated IL-31 levels may benefit from such targeted treatments.

### 3.4. Alopecia Areata

Alopecia areata (AA) is an autoimmune disorder characterized by the sudden onset of hair loss in localized or widespread areas on the scalp, face, or body. Understanding the biomarkers associated with alopecia areata is crucial for improving diagnosis, developing targeted therapies, and monitoring treatment responses.

Higher levels of lipocalin-2, insulin, and c-peptide [28] have been revealed in patients with AA, confirming the complex interconnections between an underlying inflammatory state and potential involvement of insulin resistance. These findings underscore the multifaceted nature of AA, where not only inflammatory markers like lipocalin-2 but also insulin-related and metabolic factors contribute to the intricate web of interactions that influence the development and progression of this autoimmune hair loss disorder. A recent meta-analysis indicated significant associations for TNF-α, IL7a, IFN-γ, C-reactive protein, IL-6, and vitamin D with AA [29]. The increase in these markers suggest an inflammatory milieu, implicating these factors in the autoimmune attack on hair follicles. A significant correlation between the CTLA-4 rs231726 polymorphism and AA susceptibility has also been found [30], which is indicative of mutations in the development of peripheral tolerance and autoimmunity, as well as a significant correlation between the PTPN22 rs2476601 polymorphism (the most common single-nucleotide polymorphism of the gene) and AA [31]. Such genetic markers can help identify inheritance and at-risk populations, along with the development of targeted therapies, for earlier treatment of hair loss.

With several cytokines implicated in the pathogenesis of AA and JAKs being integral in transmitting signals from these cytokines, JAK inhibitors exhibit potential in addressing AA. Ritlecitinib and brepocitinib, inhibitors of JAK3 and JAK1, respectively, showed a transcriptomic expression shift in the alopecia areata-affected scalp toward a healthier, non-lesional profile [32]. Studies also reveal the efficacy of JAK inhibitors ruxolitinib [33], tofacitinib [34], ritlecitinib [35], and baricitinib [36] in inducing hair regrowth; a systematic review found that baricitinib, ritlecitinib and brepocitinib appear to have equal efficacy for AA in clinical trials [37]. Intralesional vitamin D3 has also been determined to be an effective treatment option for localized patchy AA [38], given that high expression of vitamin D receptors (VDR) is present in keratinocytes of hair follicles; this may suggest a link between vitamin D and the development of hair follicles and epidermal differentiation.

### 3.5. Vitiligo

Vitiligo is an autoimmune depigmenting disorder of the skin due to the selective destruction of melanocytes [39]. Several different biomarkers have been identified as relating either to disease etiology or treatment response. An existing systematic review has identified the following biomarkers with vitiligo: cytokines (IL-1β, IL-17, IFN-γ, TGF-β), autoantibodies, oxidative stress markers, immune cells, and antibodies (RCLs), soluble CDs (sCD25, sCD27), and chemokines (CXCL9, CXCL10) [40].

The present search yielded additional results. First, tyrosinase (TYR) and tyrosinase-related protein-1 (TYRP1), have been identified as markers of disease etiology and treatment efficacy. In a multiple machine learning model analysis study, differentially expressed genes were identified and analyzed for functional correlation. TYR, TYRP1, Dopachrome Tautomerase (DCT), La-ribonucleoprotein-7 (LARP7), and Kinesin Family Member 1A (KIF1A) were identified as candidate biomarkers for vitiligo with associated immune infiltration [41,42]. A cross-sectional study sought to validate serum soluble CD27 (sCD27) and macrophage Migration Inhibitory Factor (MIF) using 22 active and 10 stable vitiligo patients and 32 healthy controls. Results showed significantly higher serum levels of sCD27 (*p* < 0.001) and MIF (*p* = 0.01) in vitiligo patients than in the control group. A significant positive correlation was seen for active vitiligo patients as an indicator of disease severity for both MIF (*p* = 0.002) and sCD27 (*p* < 0.001). Additional correlations were observed for clinical regression of disease and sCD27 levels [43]. MIF is known to act as a regulator of cell-mediated immunity through macrophage activation [44]. Its role as a marker may be due to the great presence of macrophages in vitiligo lesions, clearing apoptotic melanocytes [45]. sCD27 is expressed on T, B, and NK cells and is a known biomarker of immune activation. Its role in vitiligo is suggested to be due to the role of T-cells in its autoimmune destruction [43,46]. Additional existing studies outside of our search further support the role of MIF and sCD27 as biomarkers [47,48,49].

Alarmins are cellular proteins with chemotactic and immune activating properties, released in response to cell injury, death, or as an immune response [50]. Several additional markers, including HMGB1, S100B, IL-1α, and S100A9 are alarmins identified as having a significant association with vitiligo presence. Further, S100B, S100A9, and HMGB1 were identified to have a significant association with disease severity [51,52].

Cytokines, including IL6 and TNF-α were identified as being significantly higher in patients with vitiligo (*p* < 0.001), and IFN-γ as being significantly lower (*p* < 0.001) [53]. In this study, TNF-α did not differ in skin versus serum levels, whereas IL6 was significantly greater in the serum. MicroRNA (miRNAs) are short, noncoding RNAs involved in gene regulation. 12 specific miRNAs, involved in melanogenesis (miR-423, miR-182, miR-106a, miR-23b, miR-9, miR-124, miR-130a, miR-203a, miR-181, miR-152, and miR-320a) were identified as markers of disease presence, with miR-423 having the strongest association [54]. Additional studies outside of our search support these findings [55,56].

Raftlin, a marker of oxidative stress, was also identified as a significant biomarker in vitiligo [57]. Melanocyte destruction is believed to be due to free radical-induced oxidative stress and a subsequent rise in Raftilin levels. The existing literature on Raftlin in vitiligo is scarce, and future studies are indicated before conclusions regarding its clinical value can be made.

### 3.6. Chronic Spontaneous Urticaria

Chronic spontaneous urticaria (CSU) is a disease that presents with unprovoked wheals and angioedema caused by mast cell and basophil activation with subsequent degranulation and histamine release. Two subtypes exist that belong to either a type 1 hypersensitivity (auto-allergic) or a type IIb hypersensitivity (autoimmune) reaction [58]. A variety of biomarkers have been identified for CSU, and some key markers are summarized in Table 1. IgE, a main driver of mast cell degranulation and contributor to disease pathology, has inconsistent results regarding its levels correlating to disease activity. High levels of IgE may suggest high disease activity, longer duration of disease, increased response to omalizumab, and decreased response to cyclosporine. Low IgE, in contrast, may suggest type II disease, with poor omalizumab response and increased cyclosporine response [59]. Despite these key findings, additional biomarkers are needed to tailor personalized treatments.

A recent review identified a list of markers that can be used to define disease activity, including CD203, C-reactive protein (CRP), lipocalin-2 (LCN2), prothrombin fragment 1+2, D-dimer, and other cytokines including IL-6, IL-17, IL-23, TNF-α. Disease course and severity can be monitored with IL6, anti-TPO [60,61]. Specifically, anti-TPO antibodies were associated with increased severity and longer disease duration [56]. Additional markers include autologous serum stem test (ASST) positivity, which measures for autoantibodies against FcεRI, and basophil FcεRI expression. Such markers often show disease presence and can correlate with disease activity. IgE and CRP were found as markers of disease presence and activity and were also linked to distinct miRNA profiles [61,62,63]. In one study, high CRP and miR-221 levels suggest an autoimmune etiology with a prominent role of IL-31 as a marker of disease presence and severity [59]. There are some studies, however, that provide conflicting results, thus complicating the reliability of these biomarkers to be fully integrated into clinical practice. In one study, no correlation was found between PT, aPTT, D-dimer, CRP, C3, and disease activity [64].

Current standard treatments include second-generation oral antihistamines (sgAHs), omalizumab, and cyclosporine [65]. Several of these antibodies were identified as markers of treatment response to various treatments, including omalizumab, sgAHs, and cyclosporine (CSA). Concomitant anti-FcεRI IgG and IgE autoantibodies were found to be a marker seen only in late responders and poorer responders to omalizumab [66]. IL-31 was determined to decrease in response to omalizumab, suggesting treatment efficacy [67], and vascular markers such as D-dimer had inconsistent results regarding the efficacy of omalizumab, in some studies, it paralleled levels following treatment, and others it was not consistent [68,69,70]. D-dimer was also found to parallel treatment response to cyclosporine [71], and high D-dimer level was associated with resistance to antihistamine therapy [72]. Regarding total IgE, it was found to predict treatment response to antihistamines, omalizumab, and cyclosporine [73]. Low levels of IgE constitute poor treatment response to omalizumab, given its mechanism of action as binding to IgE, thus lowering its free availability. Markers of basophil activity, such as CD203 and CD63, can also suggest disease activity and predict refractory treatment response to omalizumab and antihistamines [62,74]. Conversely, high levels of CRP were found to show poor treatment response to sgAHs and omalizumab, and good treatment response to cyclosporine [75,76,77]. Another therapeutic option, autologous whole blood injection (AWBI), has been controversial but shown some efficacy in treating CSU. Total IgE, D-dimer, basophil FcεRI, and CD63 were found to be potential markers of this treatment efficacy [78].

### 3.7. Acne

Acne is a common skin condition that occurs when hair follicles become clogged with oil and dead skin cells. When the hair follicles become clogged with oil and dead skin cells, it creates an environment where bacteria can thrive. The body’s immune response to this bacterial overgrowth leads to inflammation, which manifests as redness, swelling, and tenderness around the affected area. It typically presents as pimples, blackheads, whiteheads, or cysts, most commonly appearing on the face, neck, chest, back, and shoulders. Hence, it is characterized through cystic and inflammatory lesions.

Defective regulation of stem cells is considered fundamental to acne pathogenesis. Stem cells expressing LRIG1, located in the junction zone (JZ) of hair follicles, play a role in maintaining the balance of sebaceous glands, and key pathways governing stem cells, such as Wnt/β-catenin signaling [79], disrupt the normal fate determination of LRIG1-expressing cells. Activation of Wnt signaling has been shown to form cysts in the JZ, accompanied by sebaceous gland atrophy. These cysts exhibit strong expression of stem cell markers and have shown partial reduction upon treatment with all-trans retinoic acid [80].

In addition to Wnt, macrophages also induce AKT/β-catenin-dependent Lgr5+ stem cell activation and hair follicle regeneration through TNF signaling [81]. Insulin and insulin-like growth factor 1 (IGF-1) also have been shown to activate PI3K/Akt cascade, leading to an increased nuclear export of forehead box protein O1 (FoxO1). This protein is a significant contributor to acne formation, wherein it not opposes the expression of SREBP-1c and inhibits lipogenesis, but also activates the adenosine 5-monophosphate-activated protein kinase (AMPK) pathway, which in turn negatively regulates mTORC1, an essential downstream element of the PI3K/Akt pathway [82]. Macrophages are crucial in this pathway. An injury has been shown to trigger release of the chemokine CCL2 by hair follicle (HF) keratinocytes, which subsequently attracts macrophages, specifically CX3CR1 bone marrow-derived macrophages that secrete TNF and TGFβ1; TNF activates HFSCs via the AKT/β-catenin signaling axis, leading to wound-induced hair anagen cell re-entry/growth (WIHA) and wound-induced hair follicle neogenesis (WIHN), while TGFβ1 signaling is crucial for WIHA/WIHN and may promote hair follicle regeneration through the AKT/PI3K pathway [83].

Recent work also suggests that different populations of hair follicle stem cells (HFSCs) play distinct roles in sebaceous gland renewal, with upper bulge stem cells being particularly important during the hair growth cycle, and all populations being involved in wound healing [84]. Additionally, this study shows β-catenin signaling to be crucial for the contribution of HFSCs to sebaceous gland replenishment.

Transcriptome analysis has revealed IL-1β, CXCL8, toll-like receptor (TLR) 2, CXCL2, LCN2, and secretory phosphoprotein 1 [85] to be other potential biomarkers for early diagnosis of acne. Serum IL-17 has also been elevated in patients with acne [86].

Treatments targeting these markers and pathways have been used effectively for managing acne. For example, oral isotretinoin works by upregulating FoxO transcripton factors, which are important for the activation of androgen receptors, signaling pathways related to insulin and insulin-like growth factor-1 (IGF-1), β-catenin signaling, cell growth, apoptosis, and maintaining stability in stem cell populations. This pro-drug upregulates FoxOs to reduce androgen receptor activity, insulin signaling, lipid metabolism, and cell proliferation [87].

With many cytokines also implicated in acne pathogenesis, antibody treatments have been met with mixed results. Anti-IL-17A therapy has not been found to be significantly effective in inflammatory lesions in patients with moderate to severe acne [88]. However, superoxide dismutase 3 (SOD3) has been shown to suppress TLR-2 expression in sebocytes and keratinocytes, inhibiting pro-inflammatory cytokines like TNF-α and IL-1β, and reducing lipid accumulation [89].

## 4. Formation of Anti-Drug Antibodies

Many of the treatments for such inflammatory conditions involve antibodies to block pathways and cytokine production. However, patients may form anti-drug antibodies (ADAs) to these specific treatments themselves.

For example, a systematic review found that ADA formation in psoriasis patients treated with IL-23 inhibitors had incidence rates of 4.1–14.7% with guselkumab, 141–31% with risankizumab and 6.51–18% with tildrakizumab [90]. Anti-drug antibodies developed by approximately 7% of patients with AD who received dupilumab 300 mg every 2 weeks for 16 weeks in SOLO 1 and 2, two phase 3 trials [91].

In a study of patients with immune-mediated inflammatory diseases initiating infliximab therapy—a TNF-α inhibitor—ADA formation occurred in 19% of patients during the first 38 weeks of treatment, with risk factors including diagnosis of rheumatoid arthritis, lifetime smoking, infliximab monotherapy, higher disease activity, lower infliximab doses and serum concentrations, and nonadherence of more than 11 weeks [92]. Recognition of the signs of such treatment failure is imperative in the development of personalized therapies for patients.

## 5. Role of Biomarkers in Tailoring Personalized Treatment Strategies

Several common themes emerge when comparing existing biomarkers across various dermatological conditions. Proinflammatory markers, such as cytokines, are consistently observed, along with additional markers like MMP, miRNAs, and CRP in specific conditions. While these markers offer valuable insights into disease activity and progression, they often lack specificity in diagnosis and may not differentiate between different inflammatory conditions. Nonetheless, they play a crucial role in augmenting diagnosis, monitoring disease progression, and guiding treatment decisions due to their accessibility and cost-effectiveness.

Conversely, biomarkers obtained through informatics may hold scientific and laboratory value in elucidating disease etiology, aiding in diagnosis, and identifying therapeutic targets. However, they may have limited clinical utility due to challenges in obtaining them, associated costs, and insufficient evidence supporting their reliability as biomarkers.

The complexity of autoimmunity in dermatological diseases often leads to conflicting results in biomarker studies, highlighting the need for further research to elucidate their role. This complexity underscores the importance of personalized approaches, as disease etiology is often multifactorial, as evidenced by mixed biomarker results in some studies. Despite these challenges, biomarkers remain integral in advancing personalized dermatological care, offering valuable insights into disease mechanisms and guiding tailored treatment strategies.

Moreover, emerging technologies such as single-cell sequencing and spatial transcriptomics hold promise for uncovering novel biomarkers with enhanced specificity and predictive value. These advanced techniques enable a more comprehensive understanding of cellular heterogeneity and interactions within the skin microenvironment, potentially leading to the discovery of biomarkers that can better discriminate between different dermatological conditions and predict treatment responses more accurately. Continued research in this area is essential for harnessing the full potential of biomarkers in personalized dermatological care. 

## 6. Precision Medicine Approaches and Targeted Therapies

Biomarkers have an important role in dermatology’s journey towards precision medicine, having the potential to uncover tailored interventions that resonate uniquely with individual patients. This may enable treatment customization, allowing clinicians to navigate the complexities of dermatological conditions with enhanced accuracy [93].

**Figure 1:**
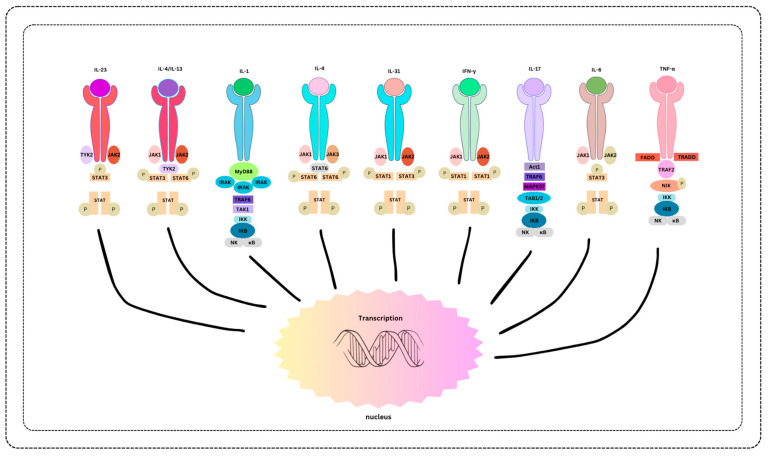
Biomarker-Associated Therapies and Treatment Modalities.

Remarkable successes in targeted therapies underscore the power of biomarker studies. There is unique potential for revolutionized treatments, such as using specific genetic markers to guide therapies in skin malignancies, leading to more effective and less invasive interventions [94]. The tailored approach, guided by biomarkers, has improved outcomes and minimized side effects, marking a paradigm shift in dermatological care [94] (Figure 1).

In the future, biomarker-driven insights can augment dermatological clinical care. From potentially harnessing AI-powered algorithms to decode biomarker patterns to exploring nanotechnology for targeted drug delivery systems, the trajectory points toward increasingly personalized and precise interventions. Biomarker research also holds promise for addressing autoimmune skin disorders, enabling more tailored immunotherapies, and possibly predicting disease progression. Moreover, integrating multi-omics data and wearable technology may allow real-time biomarker monitoring to become an integral facet of personalized dermatological care.

Additionally, the integration of multi-omics data and wearable technology holds promise for enabling real-time biomarker monitoring, allowing for more dynamic and responsive approaches to personalized dermatological care. By continuously monitoring biomarker levels and disease activity, clinicians can make timely adjustments to treatment plans, optimizing outcomes and improving patients’ overall quality of life. As the understanding of biomarkers continues to evolve, so too will the ability to deliver more precise, effective, and patient-centered dermatological interventions.

## 7. Conclusions

Biomarkers in personalized dermatological treatments represent a significant advancement, offering tailored approaches to patient care. Across a variety of conditions such as Hidradenitis Suppurativa, Psoriasis, Atopic Dermatitis, Alopecia Areata, Vitiligo, and Chronic Spontaneous Urticaria, biomarkers guide diagnosis, prognosis, treatment selection, and monitoring responses.

The success of targeted therapies driven by biomarker studies underscores their importance in improving patient outcomes. While challenges persist, the future holds promise with the integration of AI-driven algorithms, nanotechnology, and wearable technologies for real-time monitoring. With biomarkers at the forefront, dermatological care is poised for further optimization, ultimately enhancing therapeutic efficacy and improving patients’ quality of life.

## Figures and Tables

**Table 1:** Summary of Identified Biomarkers.

Condition	Biomarkers
Hidradenitis Suppurativa	TNF-α IL-1β MMPs IL-2R
Psoriasis	Interleukins and other cytokines (TNF-α, IL-23, IL-17, IL23R, IL23A) HLA markers (HLA-C06:02, HLA-B27, HLA-B38, HLA-B*08) CXCL10 Mac-2 binding protein Integrin b5 Matrix metalloproteinase-3 Macrophage-colony stimulating factor Tyramine Mucic acid LCE3D NFKBIL1 loci IgG aHDL GlycA I-FABP Kallikrein 8 Tyramine
Atopic Dermatitis	Eosinophil cationic protein Interleukins (IL-4, IL-13, IL-31) Thymic stromal lymphopoietin Filaggrin gene LDH CRP CCL17/thymus and activation-regulated chemokine (TARC), CCL26/eosinophil-attracting chemokine (eotaxin-3) CCL27/CTACK CCL18/pulmonary and activation-regulated chemokine CCL22/macrophage-derived chemokine
Alopecia Areata	Lipocalin Insulin, c-peptide Interleukins and other cytokines (TNF-α, IL7a, IFN-γ, IL-6) CRP Vitamin D CTLA-4 rs231726 polymorphism PTPN22 rs2476601
Vitiligo	TYR, TYRP1 DCT (TRP2) LARP 7 KIF1A MIF sCD27 Alarmins (HMGB1, S100B, S100A, S100A0, IL-1α) Cytokines (IL-6, TNFα, IFN-γ) Circulating miRNAs Raftilin
Chronic Spontaneous Urticaria	Total IgE IgG anti-TPO, TSH IgE to FcεRI (ASST positivity) D-dimer Interleukins and other cytokines (IL-6, IL-17, IL-23, IL-31,TNFα) CD203, CD63 CCL17 CRP S100 family (S100A8, S100A9, and S100A12)
Acne	LRIG1 IGF-1 FoxO Cytokine (IL-1β, IL-17, TNF, TGFβ1) TLR-2 CXCL2 CXCL8 LCN2 Secretory phosphoprotein 1
